# A Case of Cervicogenic Wrist Clonus Mimicking Action-Induced Tremor

**DOI:** 10.7759/cureus.91252

**Published:** 2025-08-29

**Authors:** Yoichiro Nakahara, Hisaaki Uchikado, Natsuko Miyahara, Takehiro Makizono, Motohiro Morioka

**Affiliations:** 1 Department of Neurosurgery, Kurume University School of Medicine, Kurume, JPN; 2 Department of Neurological Surgery, Uchikado Neuro-Spine Clinic, Fukuoka, JPN

**Keywords:** action-induced hand tremor, cervical spondylotic myelopathy, positional tremor, snake-eye phenomenon, wrist clonus

## Abstract

Cervicogenic wrist clonus is a rare form of action-induced hand tremor, the cases of which are not well understood. Wrist clonus from cervical spondylotic myelopathy (CSM) can be mistaken for tremor-like shaking in some patients. Here, we describe a case of a 67-year-old man who developed wrist tremors that occurred when both arms were flexed and raised. The tremor was clearly triggered by certain postures and did not respond to L-DOPA or β-blockers. The patient had no numbness or muscle weakness. A cervical MRI revealed a snake-eye phenomenon at the C3-5 levels. Computed tomography showed osteophyte formation at the C3-5 levels. Based on these findings, a localized lesion in the C6 spinal segment of the CSM was suspected, and posterior decompression surgery was performed. Tremors improved slightly after the procedure. The patient showed isolated wrist tremors without long-tract signs caused by CSM. This case highlights the importance of considering cervicogenic wrist clonus as a possible cause of tremors to ensure accurate diagnosis and timely surgical treatment.

## Introduction

Hand tremors are a common neurological symptom encountered in clinical practice. They can result from various neurological and systemic disorders, underscoring the need for precise diagnosis. Among the various types of tremor, positional tremors are relatively rare, and their mechanisms remain poorly understood [1,2]. In some cases, wrist clonus caused by cervical spondylotic myelopathy (CSM) can mimic tremor, which may lead to confusion with primary movement disorders, including Parkinson’s disease or essential tremor [3]. Surgical decompression remains the primary treatment for CSM, and timely intervention can lead to symptom improvement and help prevent further neurological deterioration. However, prognosis varies depending on factors such as the duration of symptoms and the presence of specific radiological signs. This report presents a rare case that emphasizes the importance of including CSM-related wrist clonus in the differential diagnosis of patients with atypical tremor.

## Case presentation

**Figure 1:**
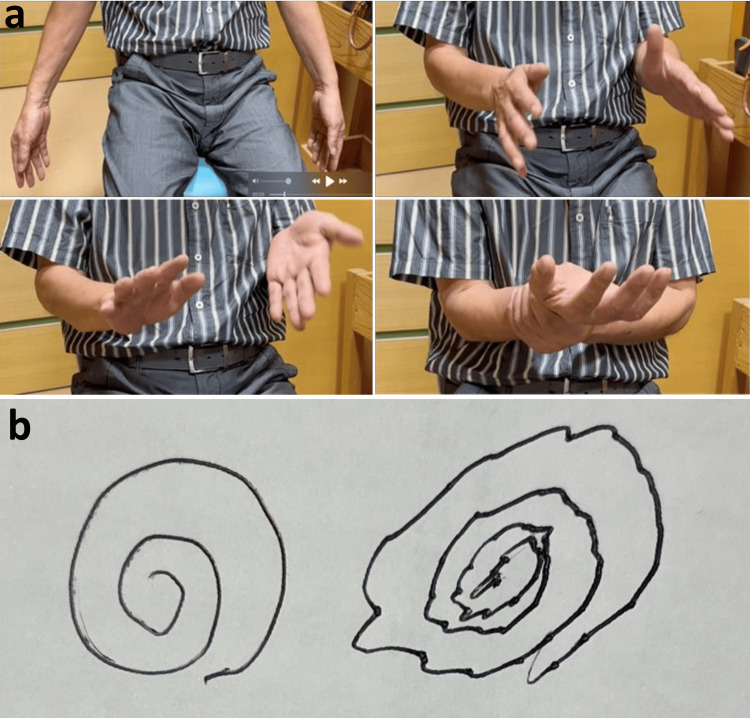
Positional tremor of the upper limb a: sequential images demonstrating posture-induced wrist clonus during bilateral arm flexion with elevation; b: handwriting trace showing tremor ring caused by posture- and action-induced wrist clonus mimicking tremor.

A 67-year-old man presented with a one-year history of hand tremor that appeared only during writing or when the arm was flexed or elevated and was absent at rest. He had no numbness, muscle weakness, or comorbidities. The tremor was initially suspected to be essential tremor and treated with β-blockers, and later Parkinsonism was considered and treated with L-DOPA; however, neither was effective. On examination, shaking tremors were observed only in specific positions (Figure 1a), and a prominent tremor ring was noted during writing (Figure 1b). Differential diagnoses, including dystonic and task-specific tremors, were also considered.

**Figure 2:**
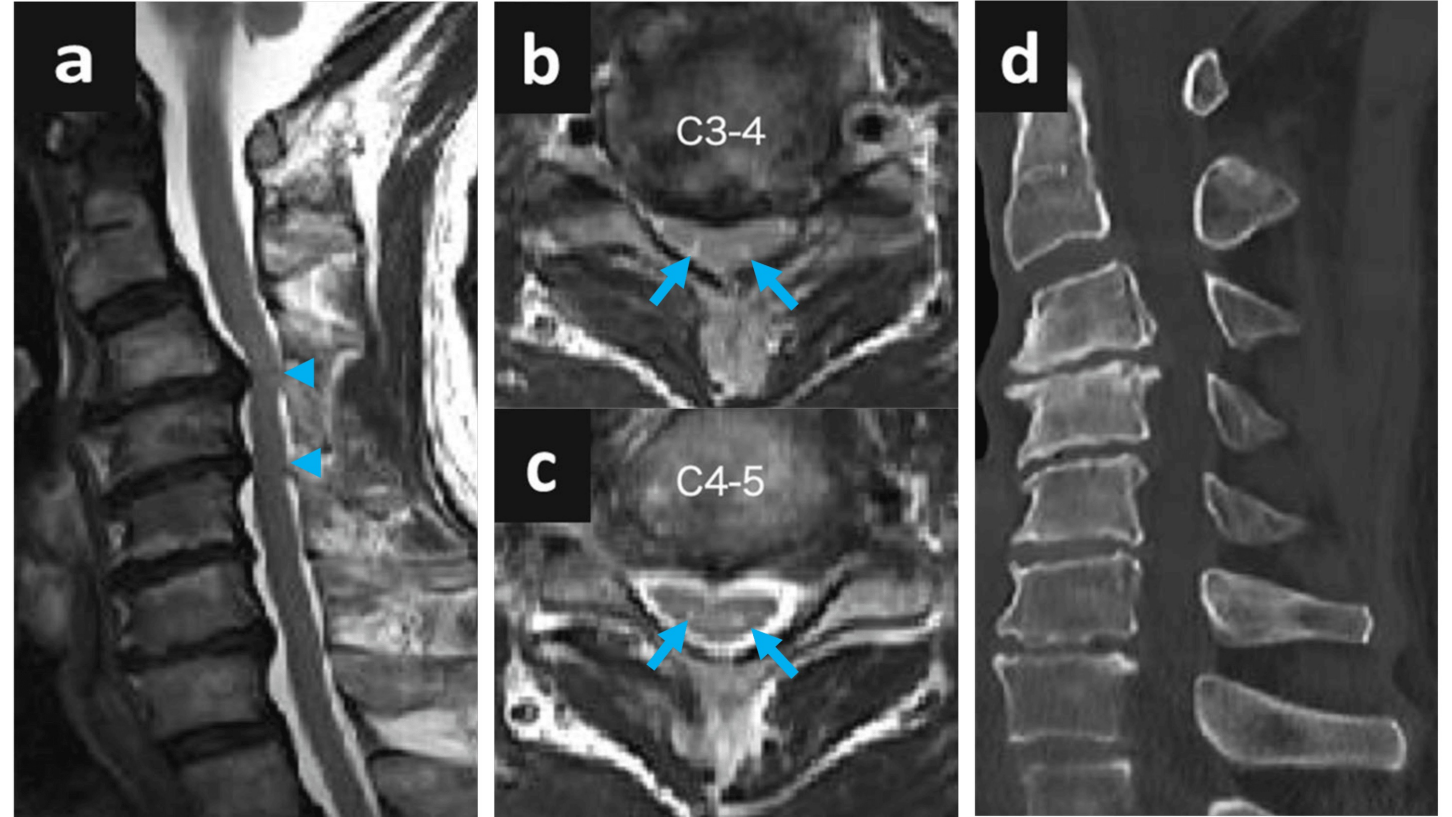
Cervical spine imaging findings a: sagittal cervical MRI showing spinal canal stenosis (arrowheads) at the C3–4 and C4–5 levels; b, c: axial cervical MRI showing “snake-eye” appearance (arrows) at the same levels; d: cervical CT showing degenerative spondylosis at the C3–4 and C4–5 levels.

Brain magnetic resonance imaging (MRI) revealed no abnormalities. Cervical MRI revealed cervical canal stenosis (Figure 2a) and spondylosis with a T2 hyperintense lesion in the anterior horn at the C3-5 level (“snake-eye” appearance) [4], corresponding to the C3-4 and C4-5 levels (Figures 2b, 2c). Computed tomography shows osteophyte formation at the same level, indicating spinal cord compression (Figure 2d). 

**Figure 3:**
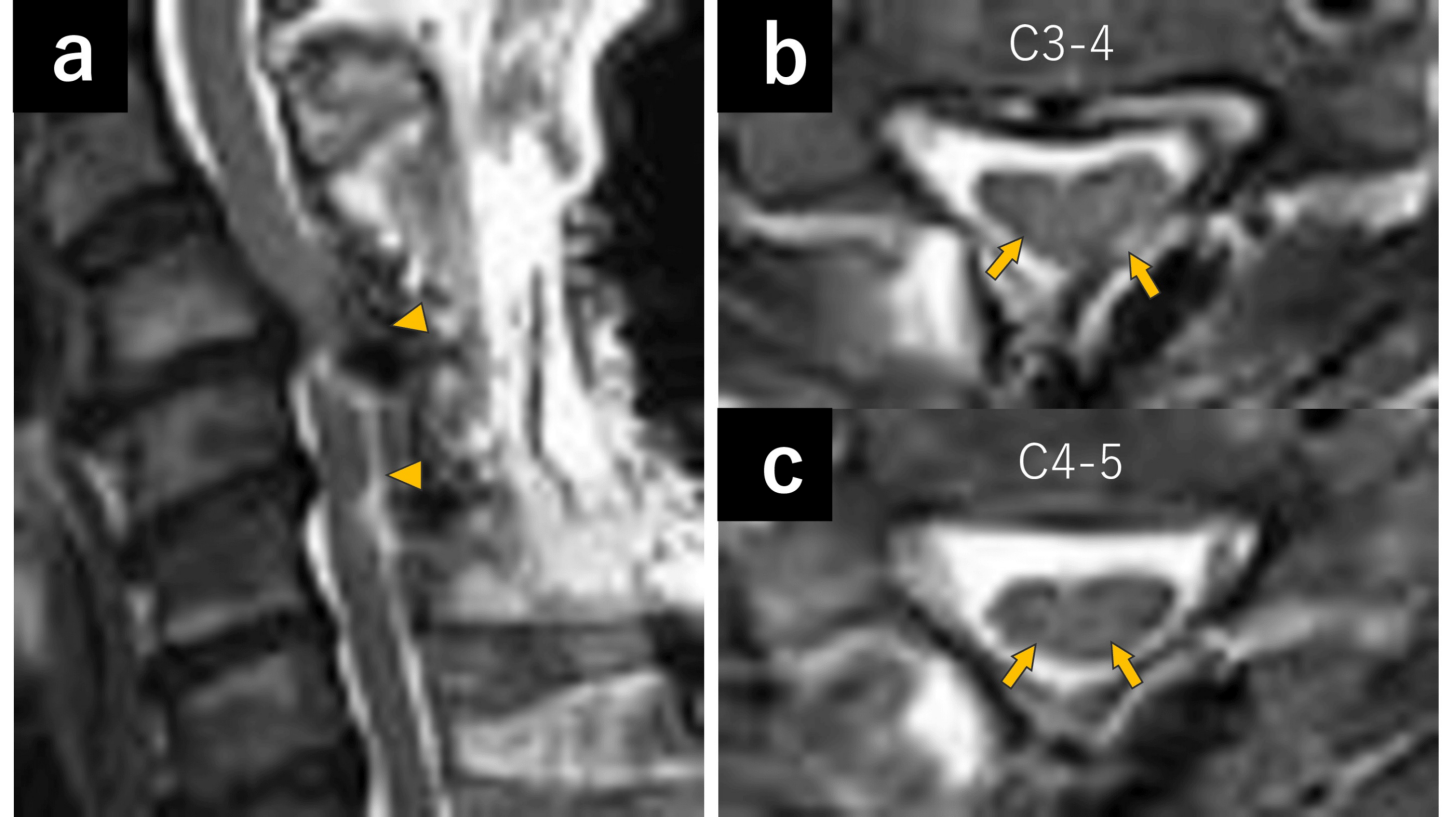
Postoperative cervical spine MRI findings a: sagittal T2-weighted MRI obtained one week after surgery showing adequate decompression at the C3–4 and C4–5 levels (arrowheads); b, c: axial diffusion-weighted MRI at the C3–4 and C4–5 levels demonstrating a slightly less distinct hyperintense area (arrows) than the preoperative images, consistent with the effects of decompression surgery.

Given the lack of improvement with conservative management and the presence of radiological findings suggestive of an increased risk of neurological deterioration, early surgical decompression was considered appropriate. A focal anterior horn lesion in the C6 segment was suspected, and posterior decompression surgery was performed (Figures 3a, 3b, 3c). At six months postoperatively, wrist shaking showed slight improvement.

## Discussion

This case represents a rare presentation of CSM manifesting as isolated, posture-dependent wrist tremor without any long tract signs. The patient initially presented with only upper limb symptoms; however, it should be noted that lower limb symptoms may develop over time, as CSM can progress with disease duration. Additionally, recurrent symptoms due to disease progression or postoperative restenosis have been reported. The presence of the ‘snake-eye’ sign is generally recognized as a poor prognostic factor in cervical myelopathy [5-7]. In this case, the symptoms persisted despite conservative management and consideration of other differential diagnoses, prompting early surgical decompression to prevent further neurological deterioration.

Fraix et al. described a case of action-induced wrist clonus resembling an action-induced tremor [8]. Wrist clonus had only been reported in a few cases and is considered extremely rare (Table 1) [9,10-17].

**Table 1:** Reported cases of tremor associated with cervical myelopathy Reported cases of wrist clonus mimicking tremor due to cervical myelopathy, illustrating its rarity. DCM: degenerative cervical myelopathy; D: disease; M: male; F: female; Bil: bilateral; L: left; NA: not available

Initial diagnosis	Author (year)	Age/Sex	Neurology	Treatment
DCM	Fraix (2008) [8]	46/M	Myelopathy	Worsening
	Sharma (2012) [10]	35/M	Bil. arm tremor	Conservative
	Magalhaes (2015) [11]	81/M	Myelopathy	Improved
	Perez (2020) [12]	39/M	Myelopathy	Improved
		57/M	L. arm tremor	Resolution
Parkinson’s D	Farris (2008) [13]	60s/M	Motor function	Improved
	Ali (2009) [14]	F	Fractures wrist	NA
		F	Myelopathy	NA
	Goh (2019) [15]	91/M	Motor function	Resolution
Hirayama D	Cerami (2008) [16]	21/M	L. arm tremor	NA
Schwannoma	Sung (2010) [17]	56/F	Myelopathy	Improved

Additionally, a recent systematic review by Khoury et al. summarized the literature on tremor as a symptom of degenerative cervical myelopathy and identified only a small number of reported cases worldwide, underscoring the rarity of this condition [9]. Davies et al. reported that tremor was observed in approximately 40% of patients with degenerative cervical myelopathy, suggesting that, although less common than other typical manifestations, it represents a clinically meaningful symptom that should not be overlooked. These data support the importance of recognizing cervicogenic tremor as a distinct clinical manifestation and considering it in the differential diagnosis. The hand tremors due to degenerative CSM are difficult to diagnose and are frequently classified into the following three groups: not diagnosed correctly, diagnosed and treated for Parkinson’s disease, and unclassifiable tremors despite a history of degenerative CSM [9]. Therefore, awareness of this atypical manifestation is essential, as prompt recognition and timely surgical intervention may prevent misdiagnosis, inappropriate treatment, and further neurological deterioration.

In this case, tremors appeared only during upper limb flexion and elevation. No sensory deficits or signs were observed in the lower limbs. The T2 hyperintense lesion matched the anterior horn at the C6 level, resembling a snake-eye appearance [4]. Damage in this area, which controls arm movements, may result in wrist clonus. Possible mechanisms include dysfunction of Renshaw cells or abnormal activation of gamma loop circuits. The involvement of an extrapyramidal system cannot be ruled out.

Cervicogenic tremors are typically accompanied by long-tract signs, including lower limb hyperreflexia. However, our patient showed wrist tremors without any signs, which is extremely rare. A compressive lesion in the corticospinal tract and impaired venous drainage may have led to the localized anterior horn lesions. While the mechanism underlying this selective involvement remains unclear, this case underscores that wrist clonus due to CSM can be mistaken for tremor [3].

Recent reports indicate that surgical intervention for CSM results in symptom improvement in approximately 60-80% of patients, although the exact rate varies depending on the evaluation scale used. For example, Galbraith et al. reported that 69% of patients showed improvement on the Nurick scale at six months postoperatively; however, only 37% maintained improvement at long-term follow-up. In contrast, the Japanese Orthopaedic Association score demonstrated improvement in 82% of patients at one year postoperatively [18]. Cervicogenic wrist clonus associated with CSM is extremely rare, and there is a paucity of data regarding postoperative outcomes; thus, the treatment success rate remains uncertain. In this case, slight symptomatic improvement was observed at six months postoperatively; however, close follow-up will remain essential to monitor for recurrence or progression.

## Conclusions

This case highlights an uncommon presentation of CSM manifesting as isolated, posture-dependent wrist tremors without any long tract signs. Such clinical features can mimic primary movement disorders, potentially delaying appropriate diagnosis. A high index of suspicion, supported by targeted neurological examination and spinal imaging, is essential for distinguishing cervicogenic wrist clonus from other tremor etiologies. This case underscores the need to consider spinal causes when evaluating patients with atypical upper limb tremors, particularly those unresponsive to standard pharmacological therapy. Identifying such conditions is critical, as timely surgical intervention may result in symptom improvement and prevent further neurological deterioration. In this case, mild symptomatic improvement was noted at six months after decompression surgery based on clinical observation; however, continued follow-up will be necessary to assess long-term outcomes and monitor for the risk of recurrence or progression.
